# Individual architecture and photosynthetic performance of the submerged form of *Drosera intermedia* Hayne

**DOI:** 10.1186/s12870-024-05155-9

**Published:** 2024-05-23

**Authors:** Krzysztof Banaś, Anna Aksmann, Bartosz J. Płachno, Małgorzata Kapusta, Paweł Marciniak, Rafał Ronowski

**Affiliations:** 1 Department of Plant Ecology, Faculty of Biology, University of Gdansk, 59 Wita Stwosza St., Gdańsk, PL 80-308 Poland; 2 Department of Plant Experimental Biology and Biotechnology, Faculty of Biology, University of Gdansk, 59 Wita Stwosza St., Gdańsk, 80-308 Poland; 3 Department of Plant Cytology and Embryology, Faculty of Biology, Institute of Botany, Jagiellonian University in Kraków, 9 Gronostajowa St., Kraków, 30-387 Poland; 4 Bioimaging Laboratory, Faculty of Biology, University of Gdansk, 59 Wita Stwosza St., Gdańsk, 80-308 Poland

**Keywords:** Aquatic plants, *Drosera intermedia*, *Drosera* habitats, Plant architecture, Chlorophyll *a* fluorescence, Photosynthetic efficiency

## Abstract

*Drosera intermedia* grows in acidic bogs in parts of valleys that are flooded in winter, and that often dry out in summer. It is also described as the sundew of the most heavily hydrated habitats in peatlands, and it is often found in water and even underwater. This sundew is the only one that can tolerate long periods of submersion, and more importantly produces a typical submerged form that can live in such conditions for many years. Submerged habitats are occupied by *D. intermedia* relatively frequently. The aim of the study was to determine the environmental conditions and architecture of individuals in the submerged form of *D. intermedia*. The features of the morphological and anatomical structure and chlorophyll *a* fluorescence of this form that were measured were compared with analogous ones in individuals that occurred in emerged and peatland habitats. The submerged form occurred to a depth of 20 cm. Compared to the other forms, its habitat had the highest pH (4.71–4.92; Me = 4.71), the highest temperature and substrate hydration, and above all, the lowest photosynthetically active radiation (PAR; 20.4–59.4%). This form differed from the other forms in almost all of the features of the plant’s architecture. It is particularly noteworthy that it had the largest main axis height among all of the forms, which exceeded 18 cm. The number of living leaves in a rosette was notable (18.1 ± 8.1), while the number of dead leaves was very low (6.9 ± 3.8). The most significant differences were in the shape of its submerged leaves, in which the length of the leaf blade was the lowest of all of the forms (0.493 ± 0.15 mm; *p* < 0.001) and usually the widest. The stem cross-sectional area was noticeably smaller in the submerged form than in the other forms, the xylem was less developed and collaterally closed vascular bundles occurred. Our analysis of the parameters of chlorophyll fluorescence in vivo revealed that the maximum quantum yield of the primary photochemistry of photosystem II is the highest for the submerged form (Me = 0.681), the same as the maximum quantum yield of the electron transport (Me φE_0_ = 0.183). The efficiency of energy use per one active reaction center of photosystem II (RC) was the lowest in the submerged form (Me = 2.978), same as the fraction of energy trapped by one active RC (Me = 1.976) and the non-photochemical energy dissipation (DI_0_/RC; Me = 0.916). The ET_0_/RC parameter, associated with the efficiency of the energy utilization for electron transport by one RC, in the submerged plant reached the highest value (Me = 0.489). The submerged form of *D. intermedia* clearly differed from the emerged and peatland forms in its plant architecture. The submerged plants had a thinner leaf blade and less developed xylem than the other forms, however, their stems were much longer. The relatively high photosynthetic efficiency of the submerged forms suggests that most of the trapped energy is utilized to drive photosynthesis with a minimum energy loss, which may be a mechanism to compensate for the relatively small size of the leaf blade.

**Supplementary Information:**

The online version contains supplementary material available at 10.1186/s12870-024-05155-9.

## Background

*Drosera intermedia* Hayne (oblong-leaved sundew) is a sundew whose name comes from the Latin *intermedius* and refers to the intermediate plant and leaf size between the small *D. rotundifolia* and the large *D. anglica*. It has a wide distribution range throughout Europe and is also present in North America and the northern part of South America [1–3], and East Africa [4]. In acidic peatlands and valley bogs, it is a plant that is primarily found in characteristic valleys and depressions where the water level is extremely high [5]. Usually, this sundew is associated with open and very wet parts of peatlands, although it is also often found on the mineral shores of lakes [6].

In Poland, *D. intermedia* is in the endangered (EN) category and has been under protection by law since 1946 [7, 8]. Like other sundews, *D. intermedia* is receding from natural habitats primarily due to their land reclamation, but also because of their uncontrolled harvesting for medicinal and ornamental purposes [5, 9, 10].

**Fig. 1:**
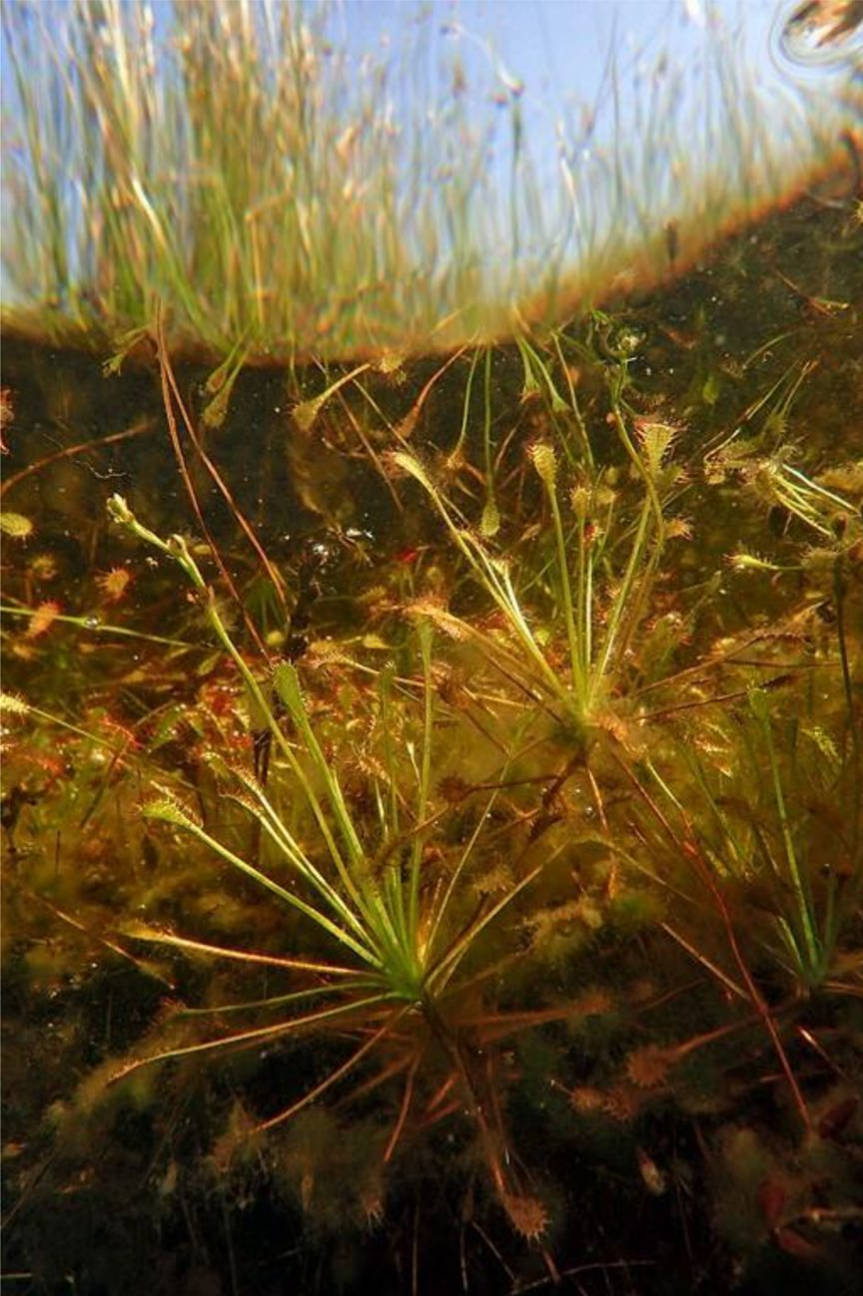
In many natural dystrophic lakes, *D. intermedia* is a permanent part of the submerged vegetation, which is usually very poorly formed there

Species of the genus *Drosera* are a rich source of bioactive substances [11–13] that can be used for medicinal purposes [14–18]. Studies have shown that *D. intermedia* extracts are more effective than the commercial products that are created from them [19], which unfortunately poses a major threat to sundews in the wild. One opportunity to improve their situation is the ever-improving in vitro culture methods for *Drosera* [20–22] including *D. intermedia* [22–24]. Relative to other species, *D. intermedia* tolerates longer periods of drought, and also total submersion (Fig. 1) although this ultimately results in a loss of its ability to capture insects.

**Fig. 2:**
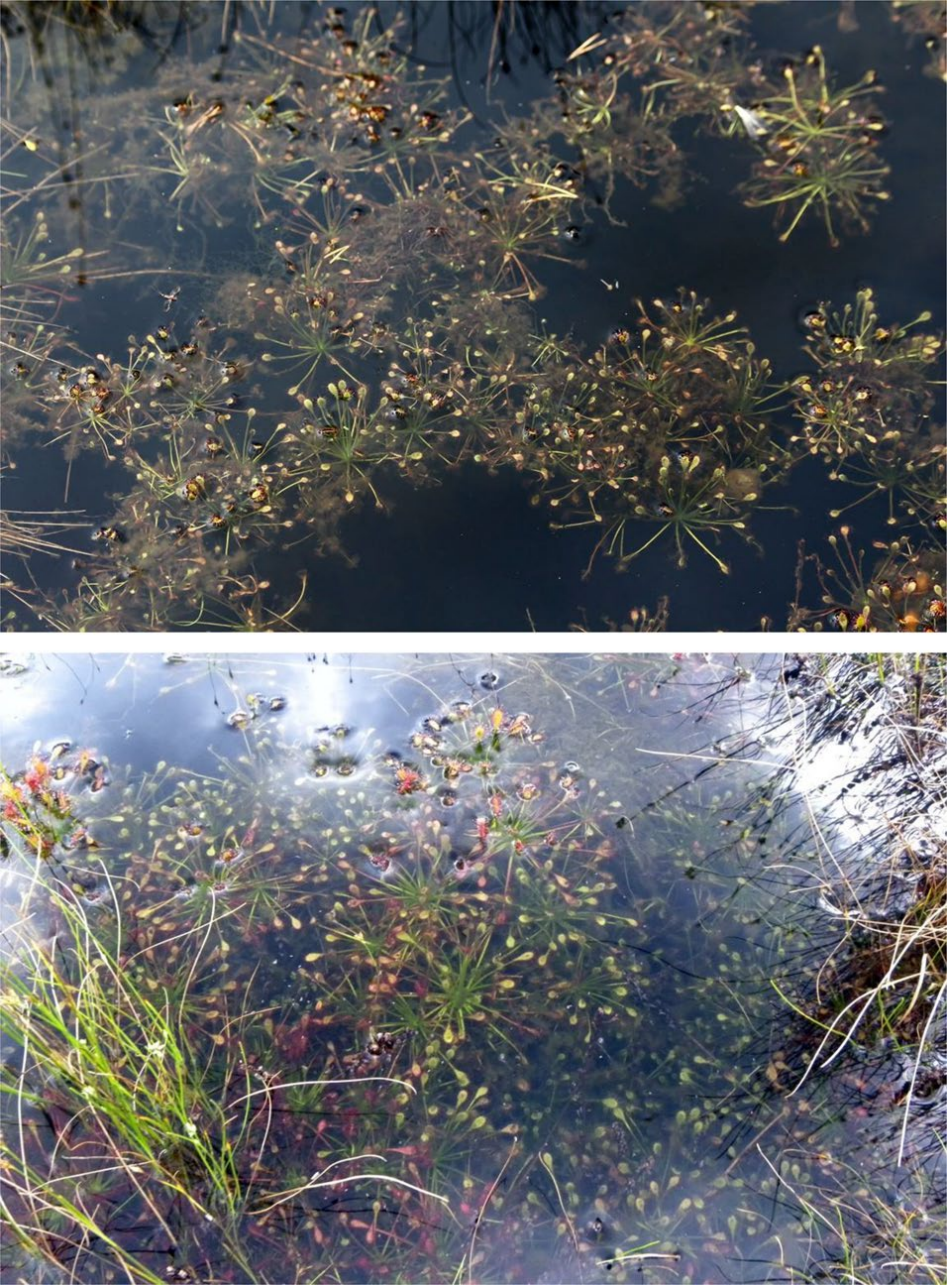
Numerous populations of the submerged form of * D. intermedia* float freely just below the surface (top photo) or are rooted in the submerged parts of the plants that form the floating mats of sphagnum in dystrophic lakes (bottom photo)

The purpose of this work was to compare the environmental conditions and architecture of individuals of this unique submerged form of oblong-leaved sundew with those that are found in other habitats within peatlands. Submerged habitats are occupied by *D. intermedia* relatively frequently, and their populations are quite numerous in some places (Fig. 2). The prerequisite for the occurrence of this form is the presence of dystrophic lakes and/or smaller ponds in peatland with a characteristic mossy blanket overlying the surface of the water. Natural dystrophic lakes are specific habitats protected under the European Natura 2000 network (habitat number 3160). These water bodies are very acidic and poor in plant nutrients. Their water has a high humic acid content and is usually stained dark brown through exposure to peat. The unique morphometric conditions of these dystrophic lakes as well as the physicochemical conditions of their waters [25–27] force the plants to adapt to very specific habitats, which are characterized by an extremely low level of nutrients, low light intensity, limited oxygen and carbon dioxide availability, etc. Therefore, in addition to determining the morphological structure of the sundews, cross sections of the stems and leaves were taken to compare the structure of the plants in different habitats, and the photosynthetic efficiency of plants was analyzed in order to determine the condition of the plants.

## Results

### Environmental conditions in *D. intermedia* habitats

**Fig. 3:**
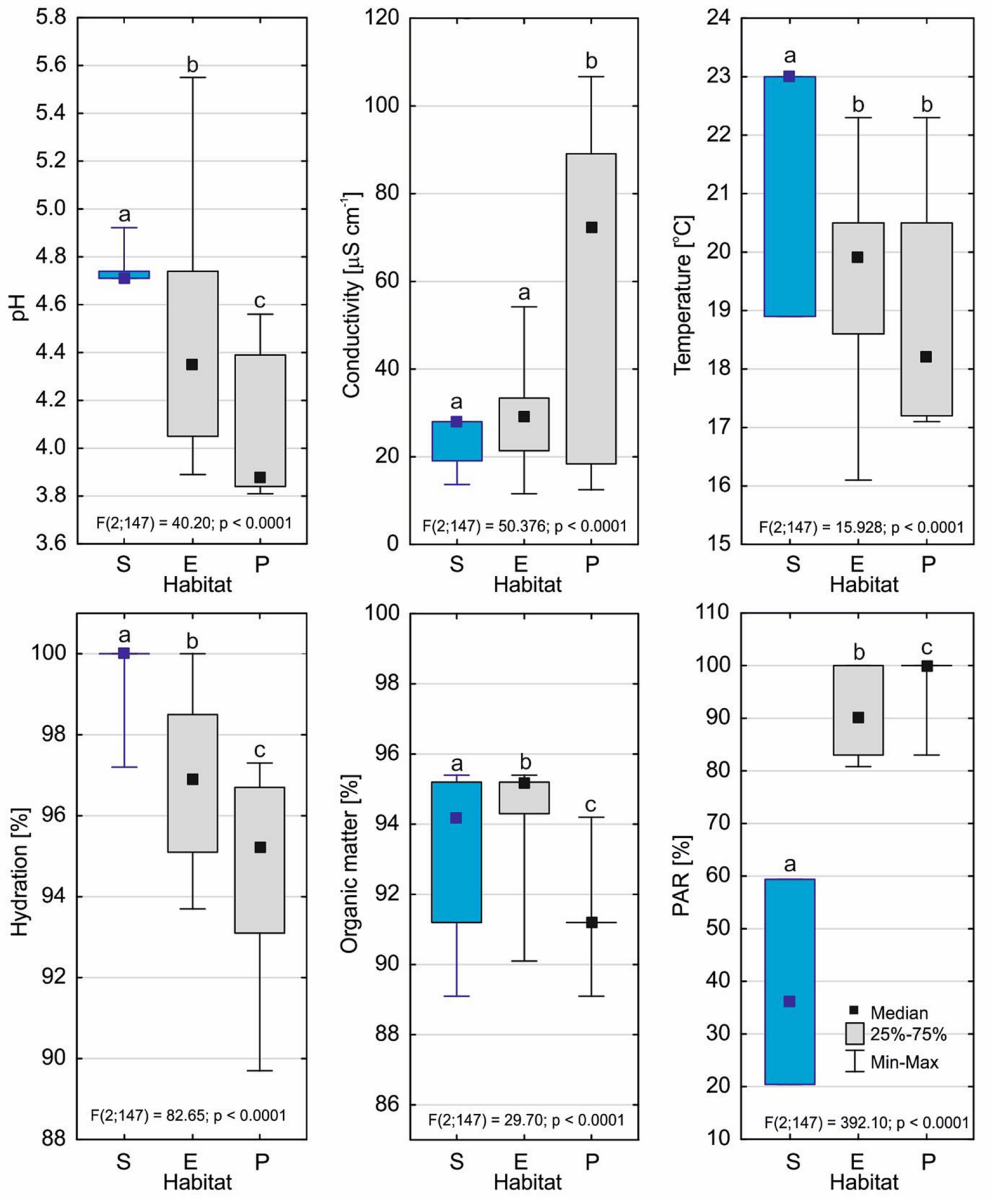
Environmental conditions of the *D. intermedia* habitats (S – submerged, E – emerged and P – peatland). Different letters (a, b, c) represent significant differences at a *p* < 0.05 probability level according to the RIR Tukey’s post hoc test using the Compact Letter Display (CLD) methodology

The submerged form of *D. intermedia* occurred in unique habitats within the studied peatbogs. The submerged habitats statistically differed in the studied traits from other habitats (emerged and peatland). First of all, they were extremely poor in dissolved mineral substances (conductivity was 22.89 ± 5.61µS cm^− 1^), which is similar to habitats of the emerged form (29.61 ± 11.76 µS cm^− 1^) and were distinguished by nearly a three-fold lower conductivity than habitats in peatlands (*p* < 0.001; Table A. 1). Moreover, they were characterized by the highest pH (*p* < 0.001; 4.71–4.92; Me = 4.71; Table 1) as well as the highest temperature and substrate hydration (*p* < 0.001), but the lowest PAR (*p* < 0.001; Fig. 3) by far. Depending on the depth of the immersion of the plants, the PAR ranged from 20.4 to 59.4% (37.9 ± 16.5%) of what reached the water surface.

**Table 1:** Environmental conditions of the *D. intermedia* habitats (S – submerged, E – emerged and P – peatland)

Characteristics	Habitat	Mean	SD	Median	Minimum	Maximum
pH	S	-	-	4.710	4.710	4.922
	E	-	-	4.350	3.890	5.550
	P	-	-	3.881	3.810	4.560
Conductivity [µS cm^− 1^]	S	22.89	5.61	28.0	13.7	28.0
	E	29.61	11.76	29.1	11.6	54.2
	P	61.83	35.35	72.2	12.5	106.7
Temperature [^o^C]	S	21.03	2.05	23.0	18.90	23.0
	E	19.46	1.88	19.9	16.1	22.3
	P	18.92	1.92	18.2	17.1	22.3
PAR [%]	S	37.89	16.45	36.3	20.4	59.4
	E	90.00	7.76	90.1	80.8	100
	P	97.17	6.43	100.0	83.0	100
Hydration [%]	S	99.63	0.89	100.0	97.2	100
	E	97.04	2.10	96.9	93.7	100
	P	94.53	2.68	95.2	89.7	97.3
Organic matter [%]	S	93.18	2.25	94.2	89.1	95.4
	E	94.45	1.61	95.18	90.1	95.4
	P	91.35	1.51	91.20	89.1	94.2

### Individual architectures of sundews

**Fig. 4:**
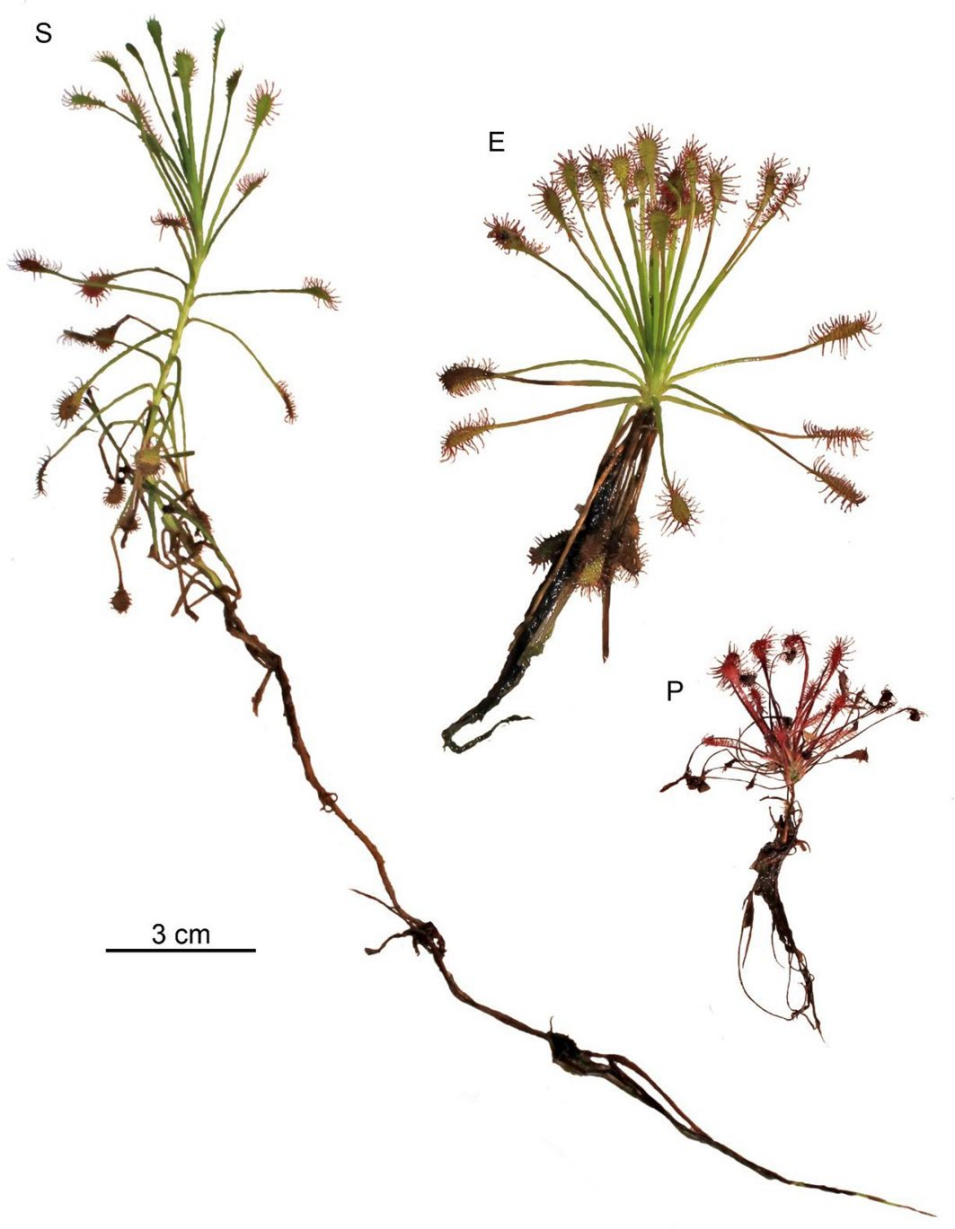
Submerged – S, emerged – E and peatland – P forms of *D. intermedia* (Peatland near Leniwe Lake – Nature Reserve Lisia Kępa)

**Fig. 5:**
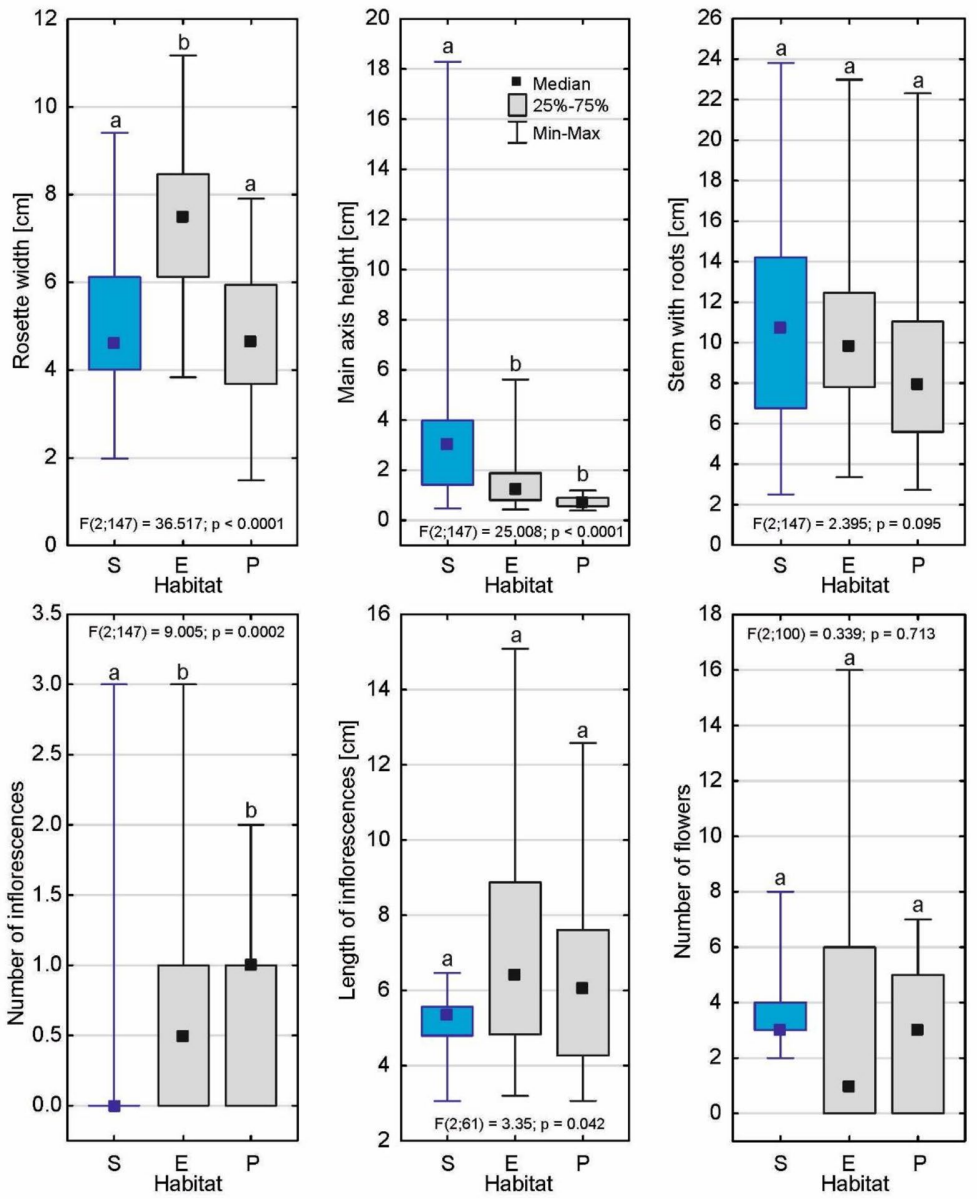
Differences in the individual architectures of the rosettes and inflorescences of *D. intermedia* from the submerged – S, emerged – E and peatland – P habitats. Different letters (a, b, c) represent significant differences at a *p* < 0.05 probability level according to the RIR Tukey’s post hoc test using the Compact Letter Display (CLD) methodology

The submerged form of *D. intermedia* clearly differed in architecture from its emerged and peatland forms in almost all of the plant characteristics (Fig. 4; Table 2), except for the length of the roots and inflorescences and the number of flowers per inflorescence (Fig. 5). The size of its rosette was 5.04 ± 1.68 cm (Me = 4.63 cm), which was significantly smaller than the aquatic form (*p* < 0.001) but similar to the peatland form (*p* = 0.87; see Additional file– Table A.1). It is worth noting that the highest main axis height among all of its forms (*p* < 0.001) was more than 18 cm. In this form, inflorescences only occurred occasionally (*p* < 0.001), and were often from the previous growing season.

**Fig. 6:**
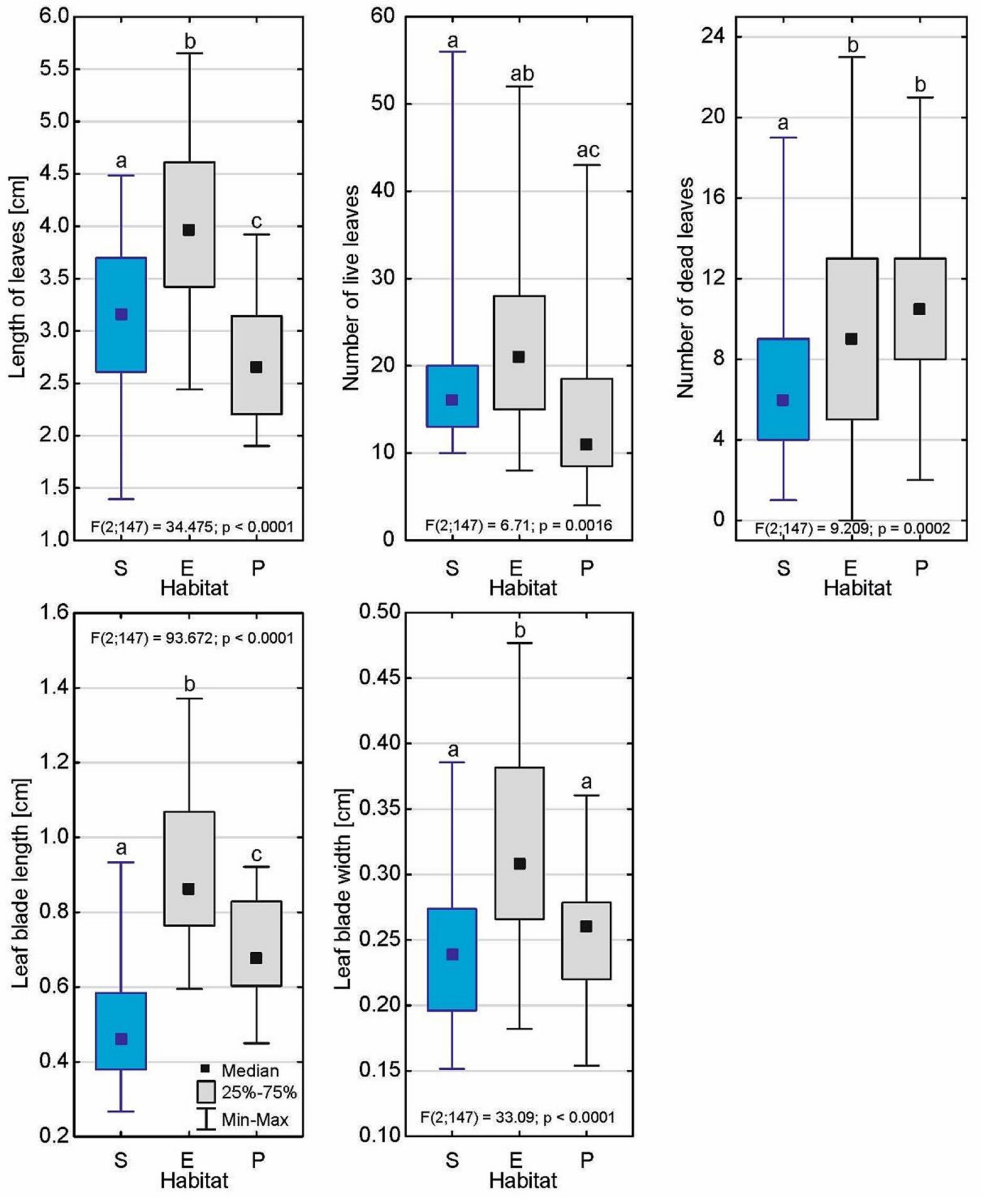
Differences in the individual leave architectures of the submerged form of *D. intermedia* (S) and the other forms (E – emerged; P – peatland). Different letters (a, b, c) represent significant differences at a *p* < 0.05 probability level according to the RIR Tukey’s post hoc test using the Compact Letter Display (CLD) methodology

**Fig. 7:**
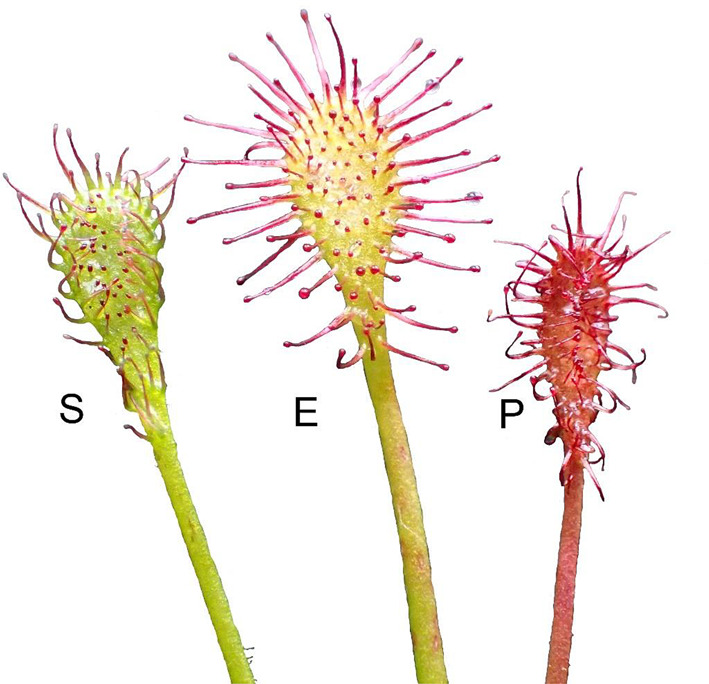
The leaves of the submerged – S, emerged – E and peatland – P forms of *D. intermedia*

**Fig. 8:**
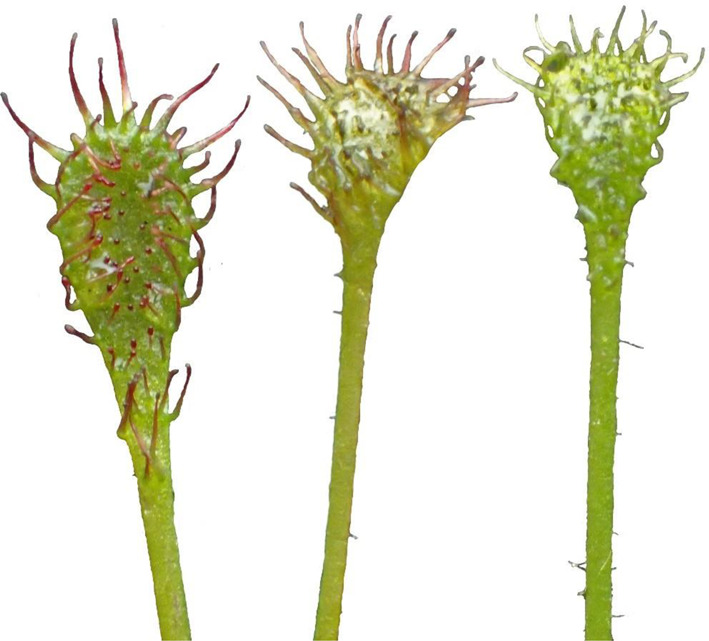
The leaves of the submerged form of *D. intermedia*

The number of living leaves was appreciable, and averaged 18.1 ± 8.1 per rosette, which was similar to the other forms (Fig. 6). The average leaf length was intermediate (3.15 ± 0.77) but lower than that found in the water form (3.98 ± 0.84; *p* < 0.001) and higher than that of the peatland form (2.71 ± 0.55; *p* = 0.035). The very low number of dead leaves (6.9 ± 3.8) as well as the appearance of the leaves themselves, which were clearly different than in the other forms was interesting (Figs. 7 and 8). Moreover, the length of the leaf blade was the lowest of all forms (0.493 ± 0.15 mm; *p* < 0.001) while the width of leaf blade was only lower than the that of the aquatic form (0.236 ± 0.048 mm; *p* < 0.001).

**Table 2:** Architecture of the sundew individuals in the different habitats (S – submerged, E – emerged and P – peatland)

Characteristics	Habitat	Mean	SD	Median	Minimum	Maximum
Rosette width [cm]	S	5.037	1.676	4.626	1.984	9.409
	E	7.366	1.742	7.510	3.838	11.171
	P	4.840	1.479	4.639	1.488	7.907
Main axis height [cm]	S	3.249	2.726	3.010	0.469	18.295
	E	1.508	0.939	1.256	0.428	5.615
	P	0.722	0.187	0.711	0.388	1.193
Stem with roots [cm]	S	10.800	5.117	10.714	2.507	23.817
	E	10.473	3.862	9.851	3.349	22.988
	P	8.780	4.402	7.985	2.726	22.312
Number of inflorescences	S	0.267	0.578	0	0	3
	E	0.685	0.843	0.5	0	3
	P	0.861	0.723	1.0	0	2
Length of inflorescences [cm]	S	5.120	1.034	5.339	3.059	6.464
	E	7.305	2.977	6.438	3.197	15.086
	P	6.317	2.545	6.076	3.064	12.578
Number of flowers	S	3.692	1.548	3.0	2	8
	E	2.963	3.650	1.0	0	16
	P	2.917	2.442	3.0	0	7
Number of live leaves	S	18.067	8.065	16	10	56
	E	21.648	8.860	21	8	52
	P	14.806	9.797	11	4	43
Number of dead leaves	S	6.883	3.792	6.0	1	19
	E	9.426	5.272	9.0	0	23
	P	10.639	4.022	10.5	2	21
Length of leaf [cm]	S	3.146	0.768	3.165	1.394	4.484
	E	3.978	0.837	3.962	2.442	5.652
	P	2.708	0.547	2.649	1.902	3.922
Leaf blade width [cm]	S	0.236	0.048	0.239	0.152	0.386
	E	0.320	0.070	0.308	0.182	0.477
	P	0.255	0.046	0.260	0.154	0.361
Leaf blade length [cm]	S	0.493	0.150	0.460	0.267	0.933
	E	0.914	0.194	0.862	0.595	1.371
	P	0.698	0.133	0.678	0.450	0.921

### Stem and leave anatomy

**Fig. 9:**
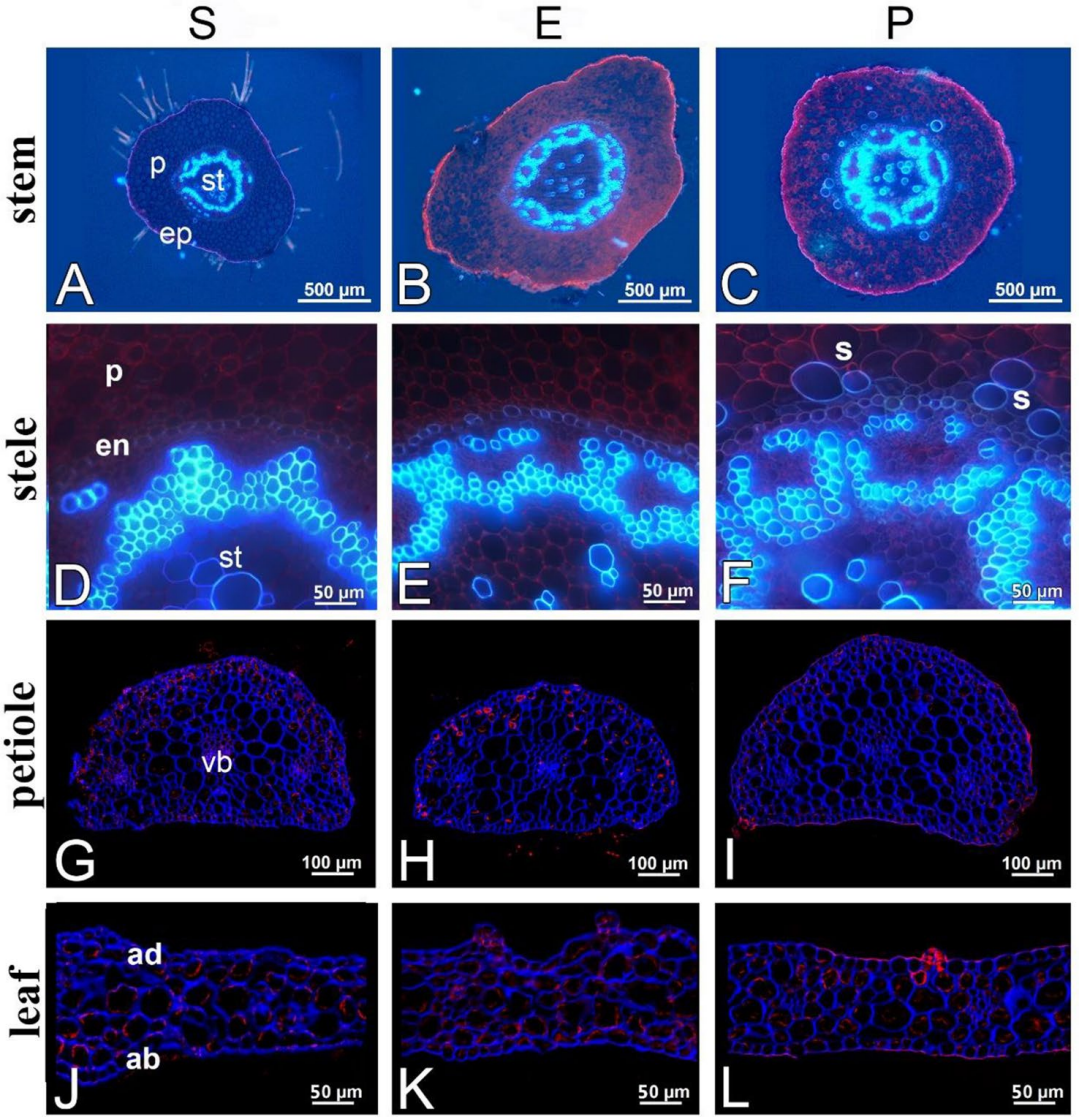
Free hand sections and cross sections of the submerged (A,D,G and J), emerged (B, E, H, and K) and peatland (C, F, I, and L) forms of *D. intermedia*. Freehand sections of the stems with a visible epidermis (ep), parenchyma (p) and stele (st). (A-F). Details of the stems and steles (st) surrounded by the endodermis (en) and parenchyma cells (p). Transverse sections of the petioles (G-I) with the major vascular bundle (vb) in the center. Transverse sections of the leaves (J-L) with the adaxial (ad) and abaxial (ab) architecture. The petioles and leaves were visualized using calcofluor and propidium iodide

In all of the plant forms, the stem consisted of a large cortex and a central cylinder (stele) with vascular bundles (Fig. 9A-F). In all of the examined plants, the cross sections revealed a primary structure (Fig. 9A-F). The epidermal cells had thin cell walls except for the external cell walls. In the submerged form, the stems were covered with filamentous algae. The stem cross-sectional area was noticeably smaller in the submerged form than in the other forms (Table 3); it is worth noting that the emerged form stood out as having the largest stem area of 1.461 ± 0.069 mm^2^. The cylinder cross-sectional area like the stem area was the smallest in the submerged form at 0.157 ± 0.017 mm^2^ and hardly varied.

The parenchyma cells of the cylinders had thin cell walls. There were small intercellulars. In the peatland form, there were many sclerenchyma cells in the internal part of the cylinder (Fig. 9F), which had thin, but lignified cell walls. In the submerged and emerged forms, there were no or only a few sclerenchyma cells in the internal part of the cylinder. The cortex and cylinder were separated by an endodermis. The endodermis cells had Casparian strips. In the emerged and peatland forms, a concentric-leptocentric type of vascular bundles occurred. In the submerged form, the xylem was less developed and collaterally closed vascular bundles occurred (but a few vessels also occurred on the outer part of the phloem).

There were no differences in the structure of the leaf anatomy (Fig. 9G-L), and the leaf blade thickness had no statistically significant differences between the studied forms, although it was the lowest in the submerged form. However, there were highly significant differences in the petiole cross-sectional area (Table 3). The submerged form was characterized by intermediate values, which were significantly higher than in the emerged form (*p* = 0.003), but lower than in the peatland form (*p* = 0.002).

**Table 3:** Stem and leaf cross-section traits of *D. intermedia* in the different habitats (S – submerged, E – emerged and P – peatland). The statistically significant differences are indicated in bold (*p* < 0.05) according to the RIR Tukey’s post-hoc test for equal abundances

Characteristics	Habitat	Mean	SD	Median	Minimum	Maximum	*p*	
							E	P
Leaf blade thickness [µm]	S	118.56	14.74	116.08	100.41	134.34	0.800	0.679
	E	126.71	29.50	118.46	101.24	172.00		0.976
	P	129.38	10.91	133.58	112.22	140.13		
Petiole cross-sectional area [mm^2^]	S	0.325	0.003	0.325	0.323	0.327	**0.003**	**0.002**
	E	0.267	0.001	0.267	0.266	0.268		**< 0.001**
	P	0.396	0.008	0.396	0.390	0.401		
Stem cross-sectional area [mm^2^]	S	0.637	0.046	0.644	0.588	0.680	**0.003**	**0.037**
	E	1.461	0.069	1.430	1.412	1.540		0.087
	P	1.097	0.282	1.087	0.820	1.383		
Cylinder cross-sectional area [mm^2^]	S	0.157	0.017	0.158	0.140	0.174	**0.034**	0.362
	E	0.402	0.109	0.382	0.305	0.520		0.218
	P	0.265	0.107	0.283	0.150	0.361		

### Photosynthetic performance

**Fig. 10:**
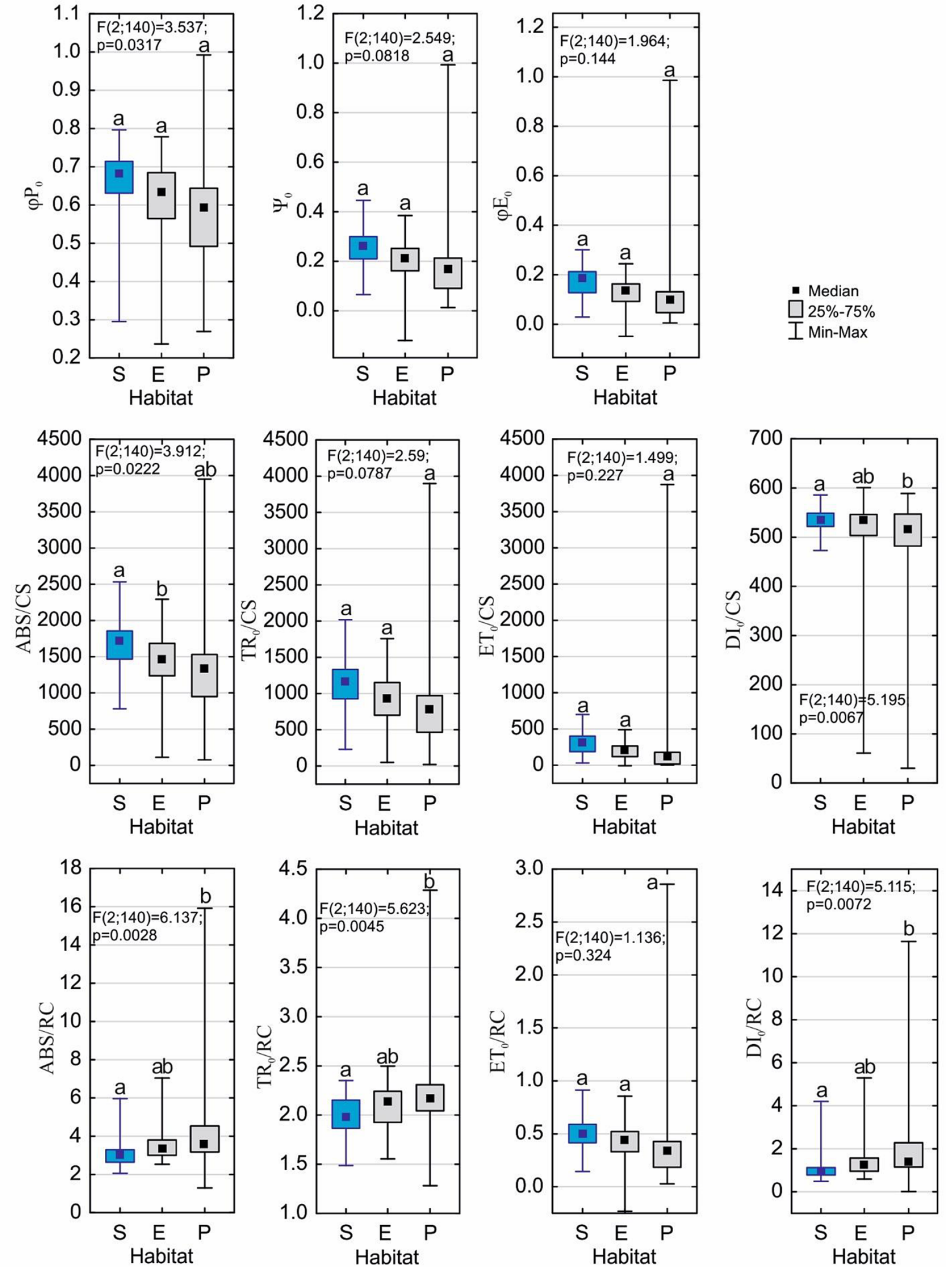
Differences in the photosynthetic parameters values of the submerged form of *D. intermedia* (S), and the other habitats (E – emerged; P – peatland). Different letters (a, b, c) represent significant differences at *p* < 0.05 probability level according to the RIR Tukey’s post hoc test using the Compact Letter Display (CLD) methodology. All of the values are given in arbitrary fluorescence units. The meanings of the abbreviations are given in the text

An analysis of the parameters of chlorophyll *a* fluorescence in vivo revealed that the maximum quantum yield of the primary photochemistry of photosystem II (φP_0_) differed significantly (*p* = 0.0317; Fig. 10, see Additional file– Table A.1) among all three forms of *D. intermedia*, and reached the highest median value for the submerged form (0.681) and the lowest one for the peatland form (0.605; Table 4; Fig. 10). The same tendency (not statistically significant, *p* = 0.144) was observed in the maximum quantum yield of the electron transport (φE_0_), which reached a median value of 0.183 in the submerged form as well as for the efficiency of the trapped exciton moving the electron further than the QA- (Ψ_0_) (*p* = 0.0818; mean value 0.260 for the submerged form).

The parameters that describe the efficiency of energy utilization per analyzed cross-section (CS) of the leaf indicated that the submerged form absorbed more light energy (median ABS/CS reached 1705.0) than the emerged and peatland forms (*p* = 0.0222; Fig. 10; Table 4). The amount of trapped energy that was absorbed by the leaf (TR_0_/CS) was also higher in the submerged form (median 1150.0) compared to the two other forms, although the effect was not statistically significant (*p* = 0.0787). A similar tendency was observed for the median value of the ET_0_/CS parameter, which describes the fraction of the energy that was utilized for electron transport by the leaves of the submerged form and reached 312.0 and was the highest among the all of the *D. intermedia* forms (*p* = 0.227). The analyzed plant forms differed significantly (*p* = 0.0067) in the efficiency of energy dissipation in a non-photochemical way (DI_0_/CS), wherein the peatland form had a much lower median value (516.0) than the submerged and emerged forms (533.0-534.0).

**Table 4:** Photosynthetic parameters characteristic for the *D. intermedia* that occupy different habitats (S – submerged, E – emerged, and P – peatland). All of the values are given in arbitrary fluorescence units. The meanings of abbreviations are given in the text

Characteristics	Habitat	Mean	SD	Median	Minimum	Maximum
φP_0_	S	0.654	0.100	0.681	0.295	0.797
	E	0.614	0.113	0.637	0.237	0.778
	P	0.590	0.145	0.605	0.269	0.992
Ψ_0_	S	0.252	0.081	0.260	0.065	0.446
	E	0.193	0.095	0.211	-0.120	0.341
	P	0.213	0.219	0.174	0.013	0.993
φE_0_	S	0.169	0.067	0.183	0.029	0.301
	E	0.125	0.064	0.139	-0.048	0.228
	P	0.143	0.204	0.104	0.005	0.986
ABS/CS	S	1636.887	357.825	1705.0	782.0	2533.0
	E	1366.148	545.847	1482.0	112.0	2292.0
	P	1366.111	780.294	1334.5	78.0	3950.0
TR_0_/CS	S	1103.415	362.870	1150.0	231.0	2018.0
	E	890.778	438.282	938.0	50.0	1759.0
	P	900.056	806.478	811.0	21.0	3899.0
ET_0_/CS	S	294.943	150.003	312.0	31.0	701.0
	E	199.704	126.982	217.0	-6.0	489.0
	P	326.306	835.983	132.0	4.0	3873.0
DI_0_/CS	S	533.472	20.505	533.0	473.0	586.0
	E	475.370	140.527	534.0	61.0	576.0
	P	466.056	160.600	516.0	30.0	601.0
ABS/RC	S	3.133	0.786	2.978	2.057	5.964
	E	3.536	0.876	3.285	2.531	7.039
	P	4.105	2.371	3.537	1.292	15.918
TR_0_/RC	S	1.979	0.215	1.976	1.487	2.352
	E	2.083	0.221	2.141	1.554	2.497
	P	2.177	0.441	2.152	1.282	4.286
ET_0_/RC	S	0.494	0.164	0.489	0.144	0.914
	E	0.401	0.193	0.436	-0.264	0.716
	P	0.469	0.558	0.343	0.028	2.857
DI_0_/RC	S	1.154	0.710	0.916	0.488	4.202
	E	1.453	0.907	1.186	0.589	5.293
	P	1.928	1.986	1.351	0.010	11.633

In the next step, the parameters that are associated with the efficiency of energy utilization per one active reaction center of photosystem II (RC) were analyzed. It was found that the ABS/RC parameters differed significantly (*p* = 0.0028) among the three *D. intermedia* forms with the lowest median value being found in the submerged form (2.978; Fig. 10; Table 4). The fraction of energy that was trapped by one active RC (TR_0_/RC) was also lowest in the submerged form (1.976) and was significantly different (*p* = 0.0045) from the parameter values that were found in the emerged and peatland forms (2.141–2.152). An opposite tendency was observed for the median value of the ET_0_/RC parameter, which is associated with the efficiency of the energy utilization for electron transport by one RC. In the submerged form, it reached the highest value (0.489), however, the differences between the plant forms were not significant (*p* = 0.324). The non-photochemical energy dissipation (DI_0_/RC) was lowest (0.916) in the submerged form and was significantly different (0.0072) from the emerged and peatland forms (Fig. 10; Table 4).

## Discussion

**Fig. 11:**
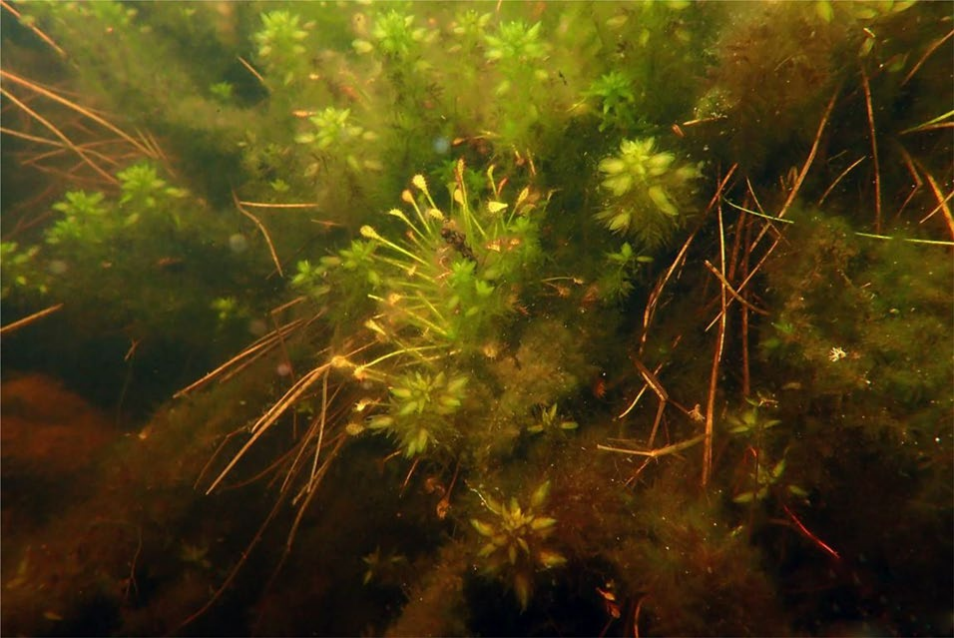
*D. intermedia* in an submerged community of *Sphagnum denticulatum* (Leniwe Lake in the “Lisia Kępa” Nature Reserve)

*Drosera intermedia* and *D. rotundifolia* generally occupy similar habitats, and they often occur together, e.g., on the mineral shores of lakes. However, *D. rotundifolia* has a much wider ecological range than other *Drosera* species and often occurs in fertile bogs such as swamp forests and swamp birches as well as in anthropogenically transformed (mainly drained) peatlands where *D. intermedia* and *D. anglica* no longer usually occur. On the other hand, *D. intermedia* is able to survive in extreme habitats, including very wet and flooded areas [5], thereby forming dense populations that float freely on the surface of the water. What is most interesting is that this sundew can live completely submersed under water. However, it should be noted that this type of submerged habitat is characteristic only for specific lake-peatland complexes, which include natural dystrophic water bodies (habitat number 3160 in the Natura 2000 network). The numerous ponds and lakes that are found here are distinguished by their specific hydrochemical conditions [26], and most importantly, by the unique structure of the banks, which are occupied by peat sloughs that overlap the water surface [27]. It is the part of the submerged environment that is primarily built by the *Carex limosa* root system and bryophytes of the genus *Sphagnum*, which hosts the submerged form of *D. intermedia*. The sundew mainly occurs here in communities of submerged bryophytes such as *Sphagnum denticulatum* (Fig. 11), *S. cuspidatum*, or *Warnstorfia exannulata*.

The environmental conditions in which the submerged form of *D. intermedia* lives stand out due to their extreme characteristics among the habitats that are occupied by this species. They were characterized by the highest pH and temperature, but extremely low conductivity, and despite significant differences from the emerged and peatland habitats, these values were within the range of variation of these characteristics. On the other hand, an essential peculiarity of the submerged habitats was the very low PAR, which varied from only 20 to 60% of the PAR that fell on the surface of the bog (see Table 1). For the other habitats where *D. intermedia* occurred, the PAR was higher than 80% (cf. Fig 1). Similar conditions of a very high PAR are found in the habitats where other *Drosera* species live [6].

Habitat flooding creates stress in plants known as either submergence stress. In nature, these stresses are important factors dictating the species composition of the ecosystem. On agricultural land, they cause economic damage associated with long-term social consequences. This contributes to breed superior cultivars to guarantee a superior yield in the face of a flooding event [28]. Flooding disrupt the movement of oxygen from the air to plant tissues [29], producing hypoxia in plants [29, 30], limits the flow of light to the plant [31], and increases the vulnerability to pathogen attack [32]. Plants respond to flooding by changes in gene expression [29, 33, 34]. These changes coordinate morphological and metabolic adaptations to the stress [33, 35]. The genes recognized as key regulators for flooding and low-oxygen tolerance were found, among others, in *Arabidopsis* [36, 37] and also in rice [38, 39], specifically in deepwater rice [40]. The specific conditions of submerged habitat caused the architecture of the submerged form of *D. intermedia* to differ from those that were found in the other habitats (cf. Figs 3 and 4) primarily by the absence of (or short) inflorescences. Further, the submerged form had a relatively low number of dead leaves, which was similar to aquatic form (live: dead ratio was about 2.5), while in the peatland form, a relatively high number of dead lives was observed (live: dead ratio was about 1.4) (cf. Table 2). It can be assumed that in aquatic and submerged environments the low number of dead leaves results from a faster decay or may be associated with the balance between the costs and benefits of leaf construction and maintenance [41] as well as the need for rapid growth in the competition for light with both other plants and the fact that they are growing up to the water surface where light conditions are much better. This last assumption is supported by the fact that stems from the fact that most of the submerged individuals were significantly elongated, which is characteristic for terrestrial plants that grow in low-light conditions or that are partially/completely submerged [42–44]. Control of shoot elongation (i.e., the elongation of leaves, petioles, and inter nodes) and adventitious root formation are plant adaptations to avoid O_2_ insufficiency under flooding stress [45, 46]. The enhanced internode elongation under flooded conditions are also indicated in *Rumex* and *Rorippa* [47]. Ethylene is the first signal to initiate these responses in plants, whereas other hormones like auxin, gibberellin, and abscisic acid and their interactions are also involved in the flooding response [48].

The adaptation of the submerged form to low light was also visible in the photosynthetic parameters. The maximum quantum yield of the primary photochemistry of photosystem II and the maximum quantum yield of electron transport reached the highest values in the submerged form and were the lowest in the peatland form (see Fig. 10; Table 4). The photosynthetic parameters, which were recalculated per analyzed cross-section of a leaf, indicated that the submerged form could absorb, trap, and utilize the light energy more efficiently than the aquatic and peatland forms could. Our study further showed that the submerged form was distinguished by the lowest efficiency of energy utilization and trapping per one active reaction center of photosystem II. However, it had the highest efficiency of the energy utilization for electron transport by one RC, which suggests that most of the trapped energy is utilized to drive photosynthesis with a minimum energy loss, which has also been suggested for submerged *Rumex palustris*, *R. crispus*, and *Phalaris arundinacea* leaves [43, 49]. Indeed, the non-photochemical energy dissipation parameters were relatively low in the submerged form (see Fig. 10; Table 4). The photosynthesis efficiency of *Hygrophila difformis* under submersion stress was investigated by Horiguchi et al. [50], who have shown that this plant acclimated to the submerged condition by increasing its photosynthetic rate. The above results suggest that submerged form of *D. intermedia* cope with submergence by means of strategy based partially on the improvement of photosynthesis underwater, as it was shown for many *Rumex*, *Ranunculus*, *Oryza* and *Scirpus* species (for review see Striker [51] and others [48, 52]).

The efficient utilization of light energy could be considered to be a mechanism to compensate for the relatively small size of the leaf blade – the trap (cf. Fig 4), which may in part explain why our results differ from the literature data [53], which has reported that the terrestrial forms of aquatic plants have smaller leaves than the individuals living in the water. It should be also considered that in many aquatic plants the reduced leaf blades of the terrestrial forms is an effective adaptive mechanism for reducing water loss through transpiration [54], which may not be entirely applicable to sundews as they occur in highly hydric habitats. In the case of submerged sundews, the production of smaller leaves could be a result of low environmental fertility and the necessity to diversify resources between growth and the stress defense [41, 55, 56].

Due to the specificity of the occupied habitat, the submerged form can also be distinguished by its anatomical structure. The submerged plants have a thinner leaf blade than the other forms, which is a common trend that is observed for plants that develop both floating and submerged leaves. For example, it was demonstrated that the floating leaves of *Potamogeton natans*, *P. polygonifolius*, *P. gramineus*, *P. zizii*, and *P. perfoliatus* were much thicker than the submerged ones [57]. The same trend was described for submerged and terrestrial leaves of *Rumex palustris* [43, 58] and *Ranunculus flabellaris* [59]. One of the reasons for the above-described changes might be a slow gas diffusion in the water, which leads to an oxygen/carbon dioxide deficiency inside the leaf that, in turn, forces changes that lead to an improved gas exchange [43, 60, 61]. Because the need for long-distance transport of water and inorganic substances in submerged plants is extremely limited, the xylem in the submerged forms was less developed than in the other forms, and there were also collaterally closed vascular bundles. In the emerged and peatland forms, there was a concentric-leptocentric type of vascular bundles. The cross-sections of the stem and leaves indicated many similarities between the submerged form and the emerged form and significant differences from the peatland form. The morphological changes and metabolic changes such as aerenchyma formation, adventitious root formation and shoot/internodes elongation are involved in plants to survive under flooded condition [62–65]. Also, aquatic leaves compared to those formed aerially can become narrower and more elongated e.g. *Potamogeton nodosus*. This can be combined to a low concentration of exogenous abscisic acid (ABA) [64].

## Conclusions

- Our work shows that among carnivorous plants, not only can species of the genera *Utricularia* and *Aldrovanda*, but *Drosera* can also be a completely aquatic plant and can survive in these conditions for a long period of time (several vegetation seasons).
- The submerged form of *D. intermedia* clearly differed from emerged and peatland forms in its plant architecture. The submerged plants had a thinner leaf blade and less developed xylem than the other forms, however, their stems were much longer. These are common adaptations found in submerged plants, indicating the high plasticity of *D. intermedia* and the persistence of its populations even in habitats with highly disturbed water levels.
- The relatively high photosynthetic efficiency of the submerged forms suggests that most of the trapped energy is utilized to drive photosynthesis with a minimum energy loss, which may be a mechanism to compensate for the relatively small size of the leaf blade. This is of great importance in the ecological success of this *Drosera* species in highly hydrated habitats, and may contribute to its persistence in flooded areas due to climate change.
- In conclusions, all of the features of the submerged form of *D. intermedia* in the analyzed material indicate that the main environmental factor that determines them is the low photosynthetically active radiation and the limited rate of gas exchange in submerged habitats. The observed adaptations of the submerged form allow us to assume that *D. inetrmedia* will be relatively resistant to climatic changes, especially rapid weather changes, as it occurs both on drained and mineral substrates, but can also occur in completely flooded organic habitats.

## Methods

### Collection of the samples and methods for analysing the environmental conditions

**Fig. 12:**
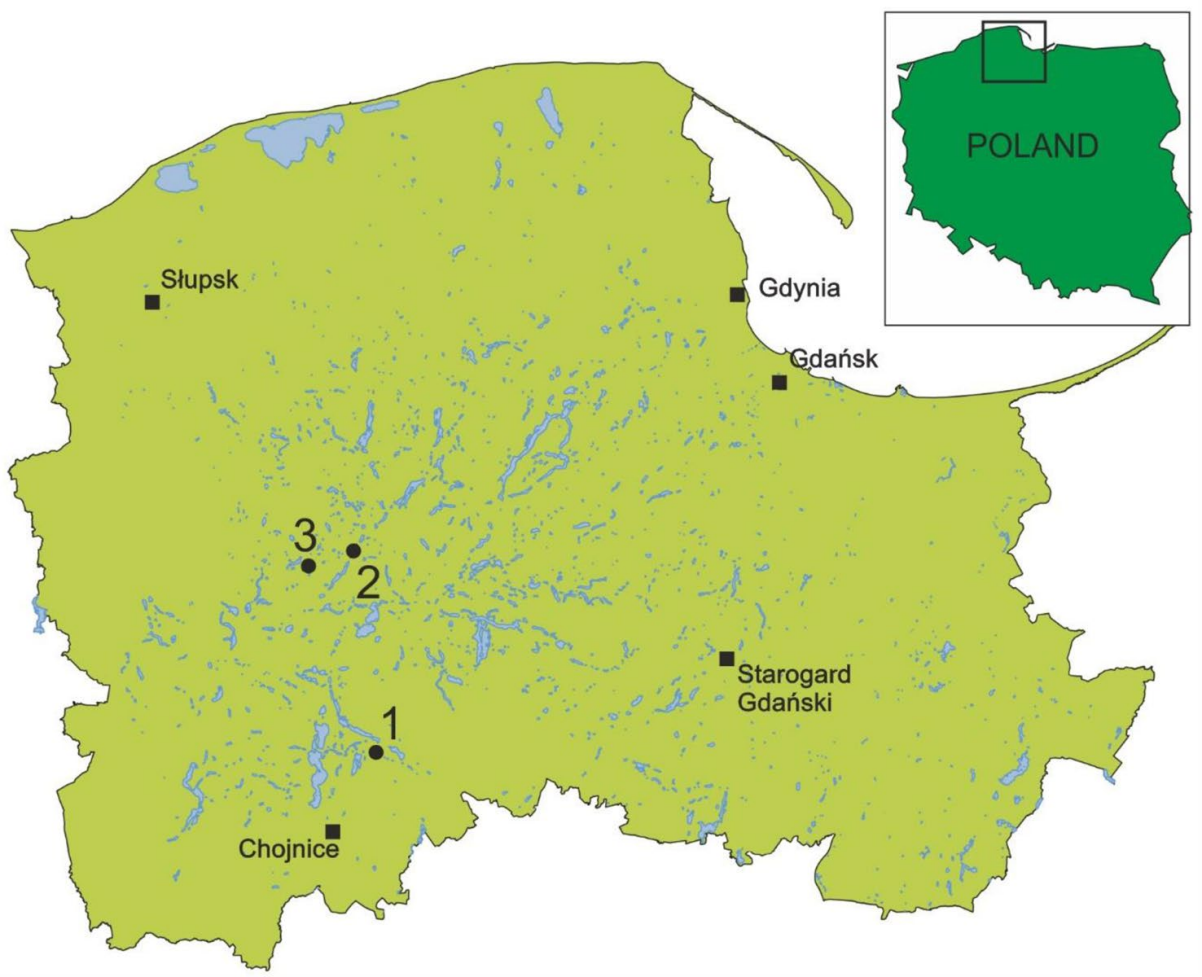
Location of the studied sites: 1- peatland near Męcikał (Zaborski Landscape Park), 2 - peatland near Studzienice (projected nature reserve), 3 - peatland near Lake Leniwe (Lisia Kępa Nature Reserve; the source of the map: Geoportal.gov.pl)

The material for the study was collected from three well-preserved high and intermediate peatlands, including one Nature Reserve in August 2022 (Fig. 12; Table 5). For the study, individuals of *D. intermedia* were collected from the three types of habitats/environments that were found at the study sites, which differ in ecological conditions:

1. submerged (where the sundew stem and leaves were completely under water);
2. emerged (sundews floated freely on the surface of the water or grew on *Sphagnum* mosses that were submerged in the water; the sundew leaves were elevated above the water, while the stem was slightly submerged or was just above the surface of the water), and.
3. peatland (sundews grew directly on the peat or on a carpet of *Sphagnum* mosses; the sundew stem and leaves were completely above the surface of the water).

**Table 5:** Coordinates of study sites (1–3). Explanations: 1- peatland near Męcikał (Zaborski Landscape Park); 2 - peatland near Studzienice (projected nature reserve); 3 - peatland near Lake Leniwe (Lisia Kępa Nature Reserve); habitat type: S - submerged, E - emerged, P - peatland (peat or a mat of *Sphagnum* mosses)

No.	Coordinates of sites	S	E	P	Total
		Number of plants			
1.	Męcikał 53°48’52.9"N 17°38’27.7"E	20	24	6	50
2.	Studzienice 54°06’25.8"N 17°34’55.8"E	10	24	6	40
3.	Leniwe 54°05’11.4"N 17°28’09.6"E	31	18	12	61
	Number of plants	61	66	24	151

The research methodology was in line with what was presented by Banaś et al. [6]. At each site, the measurement areas were designated in which the environmental conditions were determined, and the architecture of the intermediate sundew individuals was measured. For this purpose, plants were collected and the fluorescence of chlorophyll *a in vivo* was immediately measured using fluorometer for each individual. Then, all of the individuals from the site were carefully spread out on a special scaled backing and photographed for the subsequent measurements of their architectural features using a graphics program. After the photos were taken, the plants were planted in the same location from which they had been taken. Using CorelDRAW X6 from the photos, the plant architecture was measured, including primarily the rosette width, main axis height, length of roots (was usually the length of stem with adventitious roots), number of inflorescences and length of inflorescence, number of flowers, number of live and dead leaves, average length of a leaf, and the width and length of the leaf blade. The results were obtained for 151 individuals of *Drosera intermedia* using this procedure. The study was conducted after obtaining the relevant permits of the Regional Directorate for Environmental Protection in Gdansk. We adopted the species designation and nomenclature from Lowrie et al. 2017 [66]. Typical specimens of each form were preserved as herbarium material in the Herbarium Universitatis Gedanensis UGDA (herbarium number sheets: UGDA.0279037; UGDA.0279038, UGDA.0279039, UGDA.0279051, UGDA.0279052, UGDA.0279058 and UGDA.0279059), where the species identification was confirmed by Dr. Ryszard Markowski.

Using field meters, the environmental characteristics such as (1) PAR, (2) substrate temperature, (3) substrate pH, and (4) substrate conductivity were measured at all of the sites. A small sample of the substrate (about 250 ml) was collected from each site for laboratory analysis to determine its hydration and organic matter content.

The characterization of the environmental conditions at each measuring site was based on a determination of the photosynthetically active radiation (PAR), which was measured with a LI-250 Light Meter for an average of five measurements of the sundews, which was then converted for the plant site as % of light that reached the object/plant site under full illumination or that reached the water surface in the case of the submerged plants – measurements were taken at the depth of the sundews (usually 10–20 cm below the surface). The pH and temperature of the substrate were measured using a WTW 320/SET1 pH meter with a SENT IX 97T measuring electrode, while conductivity was measured using a WTW Cond 3210 SET 2 conductivity meter. Substrate hydration was calculated as a percentage from the difference in the weight between the fresh substrate and the substrate that had been dried to a constant weight at 105 °C in a Binder FD115 laboratory dryer, and the organic matter content of the substrate was calculated as a percentage of the difference in weight between the dry substrate and the substrate that had been roasted in a SEL 96 C type muffle furnace at 550 °C for five hours.

Chlorophyll *a* fluorescence in vivo was measured using a Handy Pea fluorometer (Hansatech Ltd., King’s Lynn, UK). Measurements were taken on the second or third youngest leaf of each plant. Before a measurement, the leaves were adapted to the darkness for 15 min. using dark-adaptation leaf-clips with a closed shutter-plate. Immediately prior to a measurement the shutter-plate was opened, and a saturated light pulse (10 s, 3.000 mmol photons m^− 2^ s^− 1^) was applied. The following parameters were calculated based on the fluorescence signal: the maximum yield of the primary photochemistry (φP_0_ = F_V_/F_M_); the maximum yield of electron transport (or the probability that the absorbed photon would move the electron into the electron transport chain) (φE_0_); the efficiency of the trapped exciton moving the electron further than the QA- (Ψ_0_); the specific energy fluxes per active reaction center (RC) for the energy absorption (ABS/RC), energy trapping (TR_0_/RC), electron transport driving (ET_0_/RC), and energy dissipation (DI_0_/RC); the specific energy fluxes per excited cross section of a leaf (CS) for energy absorption (ABS/CS), energy trapping (TR_0_/CS), electron transport driving (ET_0_/CS), and energy dissipation (DI_0_/CS) [67–69].

The F_V_/F_M_ ratio—recognized as a reliable measure of the photochemical activity of the photosynthetic apparatus under optimal conditions of plant growth should be approximately 0.85 relative units [70]. A decrease in the value of this parameter indicates the occurrence of stress, which can lead to the phenomenon of photoinhibition. Very low values of this parameter (about 0.2–0.3) indicate irreversible changes in the structure of PSII. However, F_V_/F_M_ was found to be highly insensitive to effects occurring under moderate drought stress in many plants grown in field conditions and was almost completely insensitive to nitrogen treatment [70]. Chlorophyll fluorescence is widely used as a plant response indicator under many abiotic constraints, but it should be remembered that it can also indicate the contribution of biotic factors such as pathogen infection and parasitism effect [71–73].

### Statistical methods that were used to develop the results

The results of both the environmental conditions and plant measurements that were obtained were summarized in a Microsoft Excel spreadsheet, where each individual was assigned the characterized environmental conditions at the site and physiological parameters, respectively. Using Statistica 13.1, the arithmetic mean and median, standard deviation, minimum and maximum value of all of the plant traits and environmental conditions were calculated. Graphs were also created to compare the habitats and traits of the different forms of sundew; the differences were determined according to ANOVA and the Tukey’s RIR post-hoc test for unequal abundances. Moreover, ANOVA and the Tukey’s RIR post-hoc test for equal abundances were used to analyze cross sections of the stems and leaves.

### Microscopy methods

Material was collected from each form of *Drosera* to make the stem and leaf cross sections. This material was fixed and preserved in 70% ethanol. Later, the stems were hand sliced using a razor blade. The sections were treated with alum carmine and iodine green and mounted in glycerol. The sections were examined using a Nikon Eclipse E400 light microscope with UV-2 A filter. The ImageJ 1.54f (Wayne Rasband and contributors, National Institutes of Health, USA) was used to distinguish the areas that were measured on the cross sections of stems and leaves that had been taken from three different but typical specimens of each form of *D. intermedia*.

Additionally, the leaves and petioles were fixed overnight in 8% (w/v) paraformaldehyde (PFA) with 0.25% (v/v) glutaraldehyde (GA) in a PIPES buffer at 4 °C, embedded in Steedman’s wax, and sectioned [74]. The chromatin of the nuclei was visualized with 1 µg/mL propidium iodide (Sigma Aldrich, St. Louis, MO, USA), and the cell walls were stained with 0.1% Calcofluor White M2R (Sigma Aldrich, St. Louis, MO, USA) [75]. The specimens were visualized using a Leica STELLARIS 5 WLL confocal microscope with the Lightning deconvolution module. The presented images are maximum projections from the Z-stacks that were taken to improve the spatial resolution.

### Electronic supplementary material

Below is the link to the electronic supplementary material.

Supplementary Material 1

## Data Availability

The datasets used and/or analysed during the current study are available from the corresponding author on reasonable request.
