# Supplementary material for: Individual architecture and photosynthetic performance of the submerged form of Drosera intermedia Hayne
Source: BMC Plant Biol. 2024 May 23;24:449. doi: 10.1186/s12870-024-05155-9 (PMC11112915; doi:10.1186/s12870-024-05155-9)
Supplement: Supplementary file 1 — Supplementary Material 1 [file 12870_2024_5155_MOESM1_ESM.docx]

**Table A1** Analysis of ANOVA of environmental, plant and photosynthetic parameters of three forms of *D. intermedia*: submerged, emerged and peatland. The differences statistically significant are marked in bold (p < 0.05)

| Traits | SS  Effect | df Effect | MS  Effect | SS  Error | df  Error | MS  Error | F | p |
| --- | --- | --- | --- | --- | --- | --- | --- | --- |
| *environmental characteristic* | | | | | | | | |
| **pH** | **10.8** | **2** | **5.38** | **19.67** | **147** | **0.134** | **40.201** | **<0.0001** |
| **Conductivity [µS cm^-1^]** | **36272.9** | **2** | **18136.45** | **52923.24** | **147** | **360.022** | **50.376** | **<0.0001** |
| **Temperature [^o^C]** | **122.1** | **2** | **61.03** | **563.20** | **147** | **3.831** | **15.928** | **<0.0001** |
| **PAR [%]** | **109936.8** | **2** | **54968.41** | **20607.64** | **147** | **140.188** | **392.105** | **<0.0001** |
| **Hydration [%]** | **600.2** | **2** | **300.09** | **533.74** | **147** | **3.631** | **82.649** | **<0.0001** |
| **Organic matter [%]** | **207.6** | **2** | **103.81** | **513.80** | **147** | **3.495** | **29.699** | **<0.0001** |
| *plant morphology* | | | | | | | | |
| **Rosette width [cm]** | **200.3** | **2** | **100.16** | **403.18** | **147** | **2.743** | **36.517** | **<0.0001** |
| **Main axis height [cm]** | **165.5** | **2** | **82.77** | **486.54** | **147** | **3.309** | **25.008** | **<0.0001** |
| Length of roots [cm] | 98.2 | 2 | 49.10 | 3013.70 | 147 | 20.501 | 2.395 | 0.0947 |
| **Number of inflorescences** | **9.3** | **2** | **4.64** | **75.69** | **147** | **0.515** | **9.005** | **0.0002** |
| **Length of inflorescences [cm]** | **43.1** | **2** | **21.53** | **392.18** | **61** | **6.429** | **3.349** | **0.0417** |
| Number of flowers | 6.4 | 2 | 3.20 | 943.45 | 100 | 9.435 | 0.339 | 0.7132 |
| **Number of live leaves** | **1037.0** | **2** | **518.50** | **11357.69** | **147** | **77.263** | **6.711** | **0.0016** |
| **Number of dead leaves** | **361.8** | **2** | **180.90** | **2887.69** | **147** | **19.644** | **9.209** | **0.0002** |
| **Length of leaf [cm]** | **38.6** | **2** | **19.32** | **82.38** | **147** | **0.560** | **34.475** | **<0.0001** |
| **Leaf blade width [cm]** | **0.2** | **2** | **0.11** | **0.47** | **147** | **0.003** | **33.090** | **<0.0001** |
| **Leaf blade length [cm]** | **5.0** | **2** | **2.52** | **3.95** | **147** | **0.027** | **93.672** | **<0.0001** |
| *plant anatomy* | | | | | | | | |
| Leaf blade thickness [µm] | 317.53 | 2 | 158.76 | 4825.63 | 12 | 402.14 | 0.395 | 0.6823 |
| **Petiole cross-sectional area [mm^2^]** | **0.0166** | **2** | **0.0083** | **0.000** | **3** | **0.0000** | **352.43** | **0.0003** |
| **Stem cross-sectional area [mm^2^]** | **1.0214** | **2** | **0.5107** | **0.173** | **6** | **0.0288** | **17.76** | **0.0030** |
| **Axial cylinder cross-sectional area [mm^2^]** | **0.0905** | **2** | **0.0452** | **0.047** | **6** | **0.0078** | **5.77** | **0.0401** |
| *photosynthetic parameters* | | | | | | | | |
| ϕ**P**_0_ | **0** | **2** | **0** | **2** | **140** | **0.0** | **3.537** | **0.0317** |
| Ψ_0_ | 0 | 2 | 0 | 3 | 140 | 0.0 | 2.549 | 0.0818 |
| ϕE_0_ | 0 | 2 | 0 | 2 | 140 | 0.0 | 1.964 | 0.1442 |
| **ABS/CS** | **2445362** | **2** | **1222681** | **43759292** | **140** | **312566.4** | **3.912** | **0.0222** |
| TR_0_/CS | 1471443 | 2 | 735721 | 39778580 | 140 | 284132.7 | 2.589 | 0.0787 |
| ET_0_/CS | 563945 | 2 | 281972 | 26333603 | 140 | 188097.2 | 1.499 | 0.2269 |
| **DI**_0_**/CS** | **145112** | **2** | **72556** | **1955500** | **140** | **13967.9** | **5.194** | **0.0067** |
| **ABS/RC** | **23** | **2** | **12** | **266** | **140** | **1.9** | **6.137** | **0.0028** |
| **TR**_0_**/RC** | **1** | **2** | **0** | **12** | **140** | **0.1** | **5.623** | **0.0045** |
| ET_0_/RC | 0 | 2 | 0 | 14 | 140 | 0.1 | 1.136 | 0.3240 |
| **DI**_0_**/RC** | **15** | **2** | **8** | **206** | **140** | **1.5** | **5.115** | **0.0072** |
